# Knowledge, Attitudes, and Practices Related to Zoster Vaccination in Riyadh First Health Care Cluster in Saudi Arabia

**DOI:** 10.7759/cureus.85743

**Published:** 2025-06-11

**Authors:** Ahmed Atiah Alzahrani, Abdulrahman Alqessayer, Khalid H Alqarni, Rayan M Almutairi

**Affiliations:** 1 Family and Community Medicine, King Saud Medical City, Riyadh, SAU; 2 Family Medicine, King Saud Medical City, Riyadh, SAU

**Keywords:** attitudes, knowledge, practices, riyadh first health care cluster, saudi arabia, varicella zoster vaccination

## Abstract

Background: Varicella zoster vaccination plays a crucial role in preventing varicella and reducing the incidence of shingles. However, varicella zoster vaccination rates in Riyadh, Saudi Arabia, remain underexplored. This study aims to address this gap by examining the factors that influence vaccine acceptance and adherence within the Riyadh First Health Care Cluster.

Objective: The primary aim is to assess the knowledge, attitudes, and practices (KAP) related to varicella zoster vaccination among the diverse population served by the Riyadh First Health Care Cluster.

Materials and methods: This cross-sectional study was conducted within the Riyadh First Health Care Cluster to assess KAP related to varicella zoster vaccination. A stratified sampling method was employed to recruit a diverse group of participants from multiple healthcare facilities. The sample size was calculated to be 264 based on a 95% confidence level, 5% margin of error, and an estimated prevalence of 22%. Data were collected through face-to-face interviews using a structured and pre-tested questionnaire covering demographics and KAP. A scoring system was applied to quantify responses. Descriptive and inferential statistics, including chi-square tests and logistic regression, were used to analyze the data. Ethical approval was obtained, and informed consent was secured from all participants.

Results: The study included 271 participants. Only 15.5% of respondents exhibited a high level of knowledge of the vaccine, while 44.3% demonstrated low knowledge, indicating a significant gap in public awareness. Despite a generally positive attitude toward vaccination, 47.6% of participants showed high acceptance, while only 18.8% reported good vaccination practices, highlighting a significant public health challenge. Notably, knowledge and attitudes were significantly related to age, gender, occupation, and income, suggesting that younger males and healthcare professionals had better awareness and acceptance.

Conclusions: The findings of this study underscore the urgent need for comprehensive public health strategies to enhance knowledge, address misconceptions, and improve vaccination uptake in Riyadh. The observed gaps in knowledge and practices, despite generally positive attitudes, highlight the importance of targeted educational initiatives that engage healthcare providers and the community.

## Introduction

Varicella zoster virus (VZV) is a double-stranded DNA virus belonging to the Herpesviridae family [1]. It is the causative agent of two distinct clinical entities: chickenpox (varicella) and shingles (herpes zoster) [2]. These diseases are characterized by their highly contagious nature and potential for severe complications, particularly in vulnerable populations such as infants, pregnant women, and immunocompromised individuals [3]. Globally, varicella causes ~4.2 million severe cases annually, with pre-vaccination incidence rates ranging from 1,000 to 1,600 per 100,000 in temperate regions. The U.S. reported a 97% decline in cases after vaccine introduction (from 4 million/year to <150,000/year) [3]. In contrast, Saudi Arabia’s pre-vaccine seroprevalence reached 90% by age 12 [3], with recent studies showing 45-60 cases per 100,000 children. For herpes zoster, the global incidence is 3-5 cases per 1,000 person-years, rising to 10-12 cases per 1,000 in adults over 80 years [4].

Varicella typically manifests with a distinctive rash, fever, and malaise, while herpes zoster presents as a painful skin rash, usually confined to one side of the body [2-4]. Following primary infection, VZV establishes latency in sensory ganglia, with potential reactivation later in life leading to herpes zoster [5]. Both varicella and herpes zoster contribute significantly to the global burden of infectious diseases, prompting the development and implementation of vaccination strategies to mitigate their impact [2]. Varicella vaccination has been incorporated into routine childhood immunization programs in various countries, resulting in a substantial decline in varicella incidence and associated complications [6]. The live attenuated varicella vaccine is highly effective in preventing severe disease and complications, with two doses recommended for optimal immunity [7]. Ongoing vaccination initiatives have significantly reduced the incidence of VZV, thereby strengthening surveillance systems, improving monitoring capabilities, and enhancing the availability and accessibility of epidemiological data [8].

The World Health Organization recognizes the importance of varicella vaccination as part of comprehensive immunization programs [9]. However, the success of vaccination efforts is contingent upon the knowledge, attitudes, and practices (KAP) of communities regarding immunization [10]. Understanding the factors influencing vaccine acceptance and uptake is crucial for designing targeted interventions and ensuring high vaccination coverage. In Saudi Arabia, the government has made significant strides in expanding its immunization programs to protect the population from vaccine-preventable diseases. The Ministry of Health in Saudi Arabia has implemented a robust vaccination schedule, including vaccines against various infectious agents [11].

Limited research has focused on the KAP related to varicella zoster vaccination in Saudi Arabia, particularly within the unique sociocultural context of Riyadh [12-15]. Factors such as religious beliefs, cultural practices, and healthcare-seeking behavior may influence the acceptance and adherence to vaccination guidelines.

Varicella vaccination has been integrated into routine immunization programs in many countries worldwide, leading to a notable reduction in varicella incidence and associated complications [6]. The live attenuated varicella vaccine has demonstrated effectiveness in preventing severe disease and complications, with two doses recommended for optimal immunity [7]. Moreover, the impact of varicella vaccination on reducing the incidence of herpes zoster highlights the broader benefits of widespread immunization [7,16]. Studies examining the KAP related to varicella zoster vaccination globally reveal a complex interplay of factors. While some regions exhibit high awareness and positive attitudes toward vaccination, challenges persist [17,18]. Misconceptions about vaccine safety, concerns over efficacy, and gaps in awareness contribute to suboptimal vaccine uptake. Cultural and religious beliefs also play a role, influencing decisions related to vaccination. Tailoring interventions to address these diverse factors is crucial for maximizing vaccine coverage [17-19]. The effectiveness of vaccination programs is closely linked to healthcare infrastructure and accessibility [6]. The availability of vaccination services, awareness campaigns, and the ease of access to healthcare facilities can influence vaccine coverage [2]. In regions with robust healthcare systems, vaccination campaigns are more likely to reach a broader population, whereas challenges in accessibility may hinder vaccine uptake [6].

In summary, while the global literature provides a foundation for understanding varicella zoster vaccination trends, a notable research gap exists within the specific context of Saudi Arabia, particularly in Riyadh. This study aims to contribute to the existing knowledge by investigating the KAP related to varicella zoster vaccination in this region, with a focus on factors unique to the local sociocultural and healthcare landscape.

## Materials and methods

Study design and sampling strategy

This study employed a cross-sectional design to collect data from a diverse sample within a specific time frame. This approach was appropriate for capturing a snapshot of KAP related to varicella zoster vaccination within the Riyadh First Health Care Cluster. A structured questionnaire was used to gather quantitative data. A stratified sampling technique was employed to ensure the inclusion of participants from various demographic groups. Stratification enhanced the representativeness of the sample, enabling subgroup analyses to explore variations in perceptions related to the vaccine. Participants were recruited from multiple healthcare facilities within the Riyadh First Health Care Cluster.

Sample size determination

The required sample size was calculated using the formula for estimating a population proportion with a specified confidence level and margin of error, assuming a 95% confidence interval (Z = 1.96), a 5% margin of error, and an expected knowledge prevalence of 22% based on a previous study in Saudi Arabia. The final sample size was 264 participants.

Inclusion and exclusion criteria

The criteria for inclusion in the study include that participants must be at least 18 years old and reside within the Riyadh First Health Care Cluster catchment area. Additionally, they should be able to comprehend the study's procedures and provide informed consent while also ensuring representation from various demographic groups, including age, gender, education, occupation, and income levels. Importantly, participants will be accepted regardless of their varicella zoster vaccination status, and fluency in either Arabic or English is required. Conversely, the exclusion criteria specify that individuals under 18 years of age, non-residents of the target area, those unable to provide informed consent, individuals with cognitive impairments that prevent participation, and those who cannot understand Arabic or English will be ineligible to participate in the study.

Data collection tool

The structured questionnaire was divided into three main sections, with each section containing 10 questions. Additionally, it gathered demographic information, including participants' age, gender, education level, occupation, and income.

For the scoring system, participants' knowledge was assessed by awarding one point for each correct answer, resulting in a maximum score of 10. Attitudes were evaluated using a 5-point Likert scale (ranging from 1 to 5), allowing for a total score between 10 and 50. Lastly, in the practice section, participants received one point for receiving the vaccine and another point for completing the recommended doses, with a maximum possible score of 2.

Data collection methods

Data were collected through face-to-face interviews conducted using the structured questionnaire. The tool was pre-tested to ensure clarity and relevance and to estimate the average time required for completion. Trained interviewers conducted the interviews to maintain consistency and data quality.

Data analysis

Descriptive statistics (frequencies, percentages, means) were used to summarize demographic characteristics and KAP variables. Inferential statistics, including chi-square tests and logistic regression, were applied to assess associations between demographic factors and KAP scores. Subgroup analyses were conducted to identify differences across demographic categories.

Pilot study

A pilot study was conducted on a small sample from the target population to assess the clarity, relevance, and feasibility of the questionnaire. Feedback from this pilot informed necessary modifications to improve the tool's effectiveness and reliability.

Ethical considerations

Ethical approval was obtained from the Institutional Review Board of King Saud Medical City (approval number: H1R1). Informed consent was obtained from all participants, with an emphasis on voluntary participation and the confidentiality of data.

## Results

Table 1 presents various demographic parameters of the participants, comprising a total of 271 individuals. The study population exhibits a younger adult dominance, with 62 (22.9%) falling within the 25-34 years age range, while 63 (23.2%) are between 45-54 years old, indicating a diverse range of perspectives regarding the research subject. The study population exhibits a significant female dominance, with 164 participants (60.5%), because gender can affect interpretations when analyzing sensitive health issues. The participant sample exhibits significant academic achievement, as 207 (76.4%) of the respondents graduated from college or university, indicating a potential awareness of the study topic. The employment diversity, combined with a high number of unemployed individuals (54; 19.9%), suggests a range of different economic circumstances that could influence the study results. The income distribution reveals substantial economic disparities among Saudi population members, as 106 (39.1%) earn an average monthly income of less than 5,000 SAR.

**Table 1:** Sociodemographic characteristics of participants (n=271) Frequencies (No.) and percentages (%)

Parameter		No.	Percent (%)
Age	18-24	56	20.7
	25-34	62	22.9
	35-44	42	15.5
	45-54	63	23.2
	55 or more	48	17.7
Gender	Female	164	60.5
	Male	107	39.5
Educational level	Primary school	9	3.3
	Secondary school	55	20.3
	College/university	207	76.4
Occupation	Healthcare professional	47	17.3
	Office worker	48	17.7
	Manual laborer	10	3.7
	Unemployed	54	19.9
	Housewife	28	10.3
	Student	34	12.5
	Retired	11	4.1
	Other	39	14.4
Monthly income	Less than 5000 SAR	106	39.1
	5000 to 10000 SAR	48	17.7
	10001 to 15000 SAR	43	15.9
	15001 to 20000 SAR	44	16.2
	More than 20000 SAR	30	11.1

**Figure 1:**
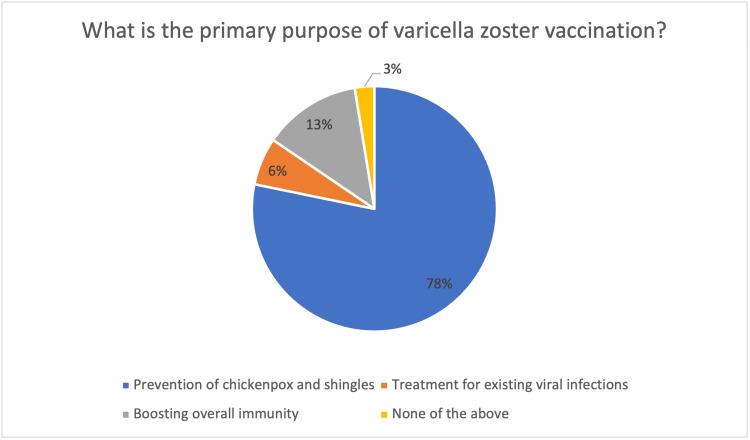
Purpose of varicella zoster vaccination according to participants Percent (%) represents the corresponding percentage of the total study population.

As shown in Figure 1, the analyzed data from 271 participants reveal essential understanding about the primary vaccination purpose among the general public for varicella zoster vaccination. Research data show that a clear majority of 212 (78.3%) successfully recognized the primary purpose of the vaccine, which is to protect against chickenpox as well as shingles, despite correctly identifying the protective benefits. An extensive minority of 17 (6.3%) misunderstood how the vaccine functions by linking it to current viral infection treatment, while another substantial minority of 35 (12.9%) thought the vaccine enhances overall immunity protection. The respondents revealed concerning knowledge gaps, as seven (2.6%) chose "none of the above" as their answer, despite the potential public health consequences this could create.

Table 2 presents key findings on how people from Riyadh perceive and understand the varicella zoster vaccination, based on a study involving 271 participants. A large percentage of 212 (78.2%) correctly recognized the vaccine's main function as protecting people from developing chickenpox and shingles, which shows good overall vaccine awareness. A significant portion of 132 (48.7%) of respondents hold the wrong belief that one lifetime dose of the vaccine satisfies the booster requirements, even though this approach may weaken long-term protection against chickenpox and shingles. The data show that pregnant women display significant concern about vaccination safety, as 161 (59.4%) of respondents expressed uncertainty about it, which requires better educational programs to provide reassurance. Only 52 (19.2%) of surveyed individuals properly identified pregnant women and immunocompromised people as vulnerable groups facing complications from the disease.

**Table 2:** Parameters related to knowledge of varicella zoster vaccination in Riyadh (n=271) * Results may overlap Frequencies (No.) and percentages (%)

Parameter		No.	Percent (%)
What is the primary purpose of varicella zoster vaccination?	Prevention of chickenpox and shingles	212	78.2
	Treatment for existing viral infections	17	6.3
	Boosting overall immunity	35	12.9
	None of the above	7	2.6
How often should adults receive a booster dose of the varicella zoster vaccine for optimal protection?	Every 5 years	75	27.7
	Every 10 years	33	12.2
	Only once in a lifetime	132	48.7
	No booster doses needed	31	11.4
Can varicella zoster vaccination be administered during pregnancy?	Yes, it is safe during any trimester	16	5.9
	Yes, but only during the first trimester	10	3.7
	No, it is contraindicated during pregnancy	84	31.0
	Not sure	161	59.4
Which population is most at risk for complications from varicella zoster infection?	Children only	14	5.2
	Older adults only	30	11.1
	Pregnant women, infants, and immunocompromised individuals	52	19.2
	Healthy adults only	175	64.6
In addition to chickenpox and shingles, varicella zoster virus can also cause:	Influenza	45	16.6
	Measles	73	26.9
	Mumps	29	10.7
	None of the above	124	45.8
What is the primary source of information for varicella zoster vaccination for you?	Healthcare professionals	93	34.3
	Media (TV, newspapers, etc.)	68	25.1
	Internet	53	19.6
	Family and friends	57	21.0
How effective is the varicella zoster vaccine in preventing shingles?	Highly effective	166	61.3
	Moderately effective	80	29.5
	Not very effective	14	5.2
	Not effective at all	11	4.1
Which age group is the varicella zoster vaccine primarily recommended for?	Children only	4	1.5
	Adults only	46	17.0
	Both children and adults	69	25.5
	Elderly only	152	56.1
Can a person who has had chickenpox in the past still benefit from the varicella zoster vaccine?	Yes	128	47.2
	No	26	9.6
	Not sure	117	43.2
What are common side effects of the varicella zoster vaccine? (Select all that apply.)*	Pain or swelling at the injection site	149	54.9
	Fever	152	56.1
	Rash	116	42.8
	None of the above	35	12.9

**Figure 2:**
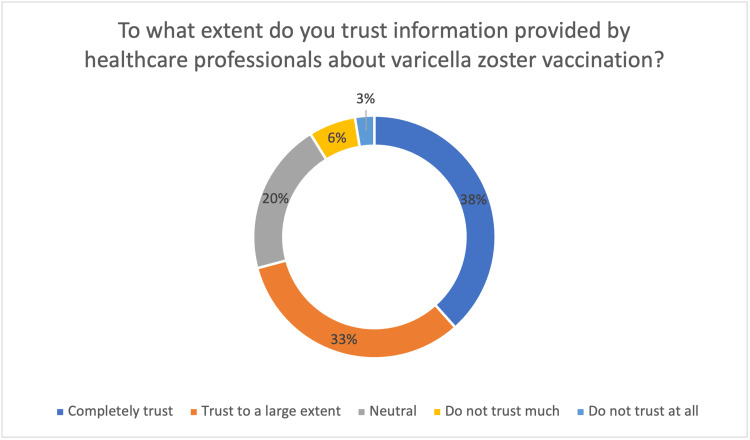
Trusting healthcare professionals about varicella zoster vaccination among participants Percent (%) represents the corresponding percentage of the total study population.

As shown in Figure 2, the analysis of trust levels regarding healthcare professionals' information about varicella zoster vaccination, based on 271 participant responses, provides a significant understanding of public perception. The majority of participants demonstrated complete trust in professional guidance, with 104 (38.4%) reaching this level and the remaining 88 (32.4%) exhibiting a high degree of trust. The survey results confirm that healthcare professionals maintain strong confidence among more than two-thirds of the respondents. Less than 10% of respondents (17, 6.3%, and 7, 2.6%) displayed mistrust toward professional information recommendations about shingles and its vaccination. Fifty-five (20.3%) respondents had neutral opinions about the information source.

Table 3 presents the results of a study involving 271 Riyadh residents who participated in an analysis of their perspectives on the varicella zoster vaccine. The vaccination's effectiveness receives considerable endorsement from interviewees since 178 (65.7%) of the total respondents demonstrated different levels of confidence. Data shows that trust in healthcare information reaches 192 (70.9%) among participants, thus demonstrating healthcare providers have the potential to impact vaccination attitudes. Patients demonstrated an understanding of the importance of vaccination discussions during check-ups, according to 184 (67.9%) respondents, indicating a need for healthcare practitioners to actively promote immunization. Community health benefits from vaccination, according to 200 (73.8%) of participants who find its impact important or extremely important, although side effect concerns exist, as shown by the 131 (48.4%) who expressed some form of worry. The data show that government health agencies need to focus their public awareness campaigns, as 205 (75.6%) of participants find these plans extremely or importantly vital.

**Table 3:** Participants’ attitude toward varicella zoster vaccination in Riyadh (n=271) Frequencies (No.) and percentages (%)

Parameter		No.	Percent (%)
How confident are you in the effectiveness of varicella zoster vaccination?	Very confident	97	35.8
	Somewhat confident	81	29.9
	Neutral	61	22.5
	Somewhat not confident	19	7.0
	Not confident at all	13	4.8
To what extent do you trust information provided by healthcare professionals about varicella zoster vaccination?	Completely trust	104	38.4
	Trust to a large extent	88	32.5
	Neutral	55	20.3
	Do not trust much	17	6.3
	Do not trust at all	7	2.6
How important do you think it is for healthcare professionals to discuss varicella zoster vaccination during routine check-ups?	Extremely important	85	31.4
	Important	99	36.5
	Neutral	55	20.3
	Not very important	24	8.9
	Not important at all	8	3.0
How concerned are you about the potential side effects of varicella zoster vaccination?	Very concerned	49	18.1
	Somewhat concerned	82	30.3
	Neutral	71	26.2
	Not very concerned	53	19.6
	Not concerned at all	16	5.9
In your opinion, how significant is the impact of varicella zoster vaccination on community health?	Extremely significant	117	43.2
	Significant	83	30.6
	Neutral	49	18.1
	Insignificant	16	5.9
	Extremely insignificant	6	2.2
How likely are you to recommend varicella zoster vaccination to family and friends?	Very likely	101	37.3
	Likely	78	28.8
	Neutral	50	18.5
	Unlikely	26	9.6
	Very unlikely	16	5.9
To what extent do you believe that varicella zoster vaccination contributes to overall healthcare costs?	Significantly contributes	111	41.0
	Contributes	78	28.8
	Neutral	61	22.5
	Does not contribute much	14	5.2
	Does not contribute at all	7	2.6
How important is it for government health agencies to promote varicella zoster vaccination through public awareness campaigns?	Extremely important	129	47.6
	Important	76	28.0
	Neutral	44	16.2
	Not very important	14	5.2
	Not important at all	8	3.0
How confident are you in the transparency of information provided by pharmaceutical companies regarding varicella zoster vaccination?	Very confident	62	22.9
	Somewhat confident	76	28.0
	Neutral	78	28.8
	Somewhat not confident	28	10.3
	Not confident at all	27	10.0
In your opinion, how well-informed is the general public about varicella zoster vaccination?	Very well-informed	33	12.2
	Well-informed	92	33.9
	Neutral	64	23.6
	Not very well-informed	68	25.1
	Not well-informed at all	14	5.2

Table 4 provides substantial details about beliefs about varicella zoster vaccination among Riyadh residents (n=271). The majority of 271 participants showed low vaccine adoption, as they had not received the varicella zoster vaccination, as reported in 228 (84.1%) of cases. Among individuals who began vaccination, only 44 (16.2%) managed to fulfill the complete dosage plan. Data shows that widespread ignorance about vaccine safety, together with fears about vaccine security, plays a significant role in this phenomenon, according to participants' responses at 63 (23.2%) and 78 (28.8%). A crucial educational effort must be launched to address participants' concerns about the necessity of the vaccine, as 91 (33.6%) participants expressed doubts about its necessity. Research data reveals concerning figures, as 205 (75.6%) of individuals neither acquire vaccine information from healthcare providers.

**Table 4:** Participants’ practice toward varicella zoster vaccination in Riyadh (n=271) * Results may overlap Frequencies (No.) and percentages (%)

Parameter		No.	Percent (%)
Have you ever received the varicella zoster vaccine?	No	228	84.1
	Yes	43	15.9
If yes, did you complete the recommended number of doses?	No	227	83.8
	Yes	44	16.2
If no, what are the reasons for not getting vaccinated? (Select all that apply.)*	Lack of awareness	63	23.2
	Concerns about vaccine safety	78	28.8
	Belief that the vaccine is unnecessary	91	33.6
	Cost	15	5.5
	Other (Specify)	84	30.9
Have you ever actively sought information about varicella zoster vaccination from healthcare providers?	No	205	75.6
	Yes	66	24.4
How frequently do you discuss varicella zoster vaccination with your healthcare provider?	Regularly	25	9.2
	Occasionally	53	19.6
	Rarely	69	25.5
	Never	124	45.8

The data presented in Table 5 elucidate the knowledge levels regarding varicella zoster vaccination among a sample population in Riyadh, revealing a concerning trend. Notably, only 42 (15.5%) of respondents demonstrated a high level of knowledge, while a significant 120 (44.3%) exhibited low knowledge of the vaccination, indicating potential gaps in public awareness and education efforts. The moderate knowledge level among 109 (40.2%) of individuals suggests that while some familiarity exists, critical misconceptions or incomplete understanding may prevail.

**Table 5:** Knowledge of varicella zoster vaccination in Riyadh score results Frequencies (No.) and percentages (%)

	Frequency	Percent
High level of knowledge	42	15.5
Moderate knowledge level	109	40.2
Low knowledge level	120	44.3
Total	271	100

The data presented in Table 6 elucidate the attitudes toward varicella zoster vaccination among a sample population in Riyadh, indicating a predominance of high and moderate positive attitudes. Specifically, 129 (47.6%) of participants demonstrated a high level of acceptance, while 113 (41.7%) exhibited a moderate attitude, collectively suggesting a favorable disposition toward vaccination initiatives. However, it is noteworthy that 29 (10.7%) of respondents were categorized as having a low attitude level.

**Table 6:** Attitude toward varicella zoster vaccination in Riyadh score results Frequencies (No.) and percentages (%)

	Frequency	Percent
High attitude level	129	47.6
Moderate attitude level	113	41.7
Low attitude level	29	10.7
Total	271	100

The findings presented in Table 7 illuminate a concerning trend regarding the practice levels toward varicella zoster vaccination in Riyadh. With only 51 (18.8%) of participants demonstrating good practices, in contrast to a striking 220 (81.2%) exhibiting poor practices, these results underscore a significant public health challenge.

**Table 7:** Practice toward varicella zoster vaccination in Riyadh score results Frequencies (No.) and percentages (%)

	Frequency	Percent
Good practice level	51	18.8
Poor practice level	220	81.2
Total	271	100

Table (8) shows that knowledge of varicella zoster vaccination has a statistically significant relation to age (p=0.019), gender (p=0.036), occupation (p=0.005), and monthly income (p=0.028). It also shows a statistically insignificant relation to educational level. Participants aged 25 to 34, male healthcare professionals, and those earning between 15001 and 20000 SAR were found to have a higher knowledge level regarding varicella zoster vaccination.

**Table 8:** Relationship between knowledge of varicella zoster vaccination in Riyadh and sociodemographic characteristics Data are represented as N, %, mean ± SD, etc., and include the value at which the p-value is considered significant (p<0.05).

Parameters		Knowledge level		Total (N=271)	χ²	p-value*
		High or moderate knowledge level	Low knowledge level			
Age	18-24	29	27	56	11.80	0.019
		19.2%	22.5%	20.7%		
	25-34	45	17	62		
		29.8%	14.2%	22.9%		
	35-44	24	18	42		
		15.9%	15.0%	15.5%		
	45-54	27	36	63		
		17.9%	30.0%	23.2%		
	55 or more	26	22	48		
		17.2%	18.3%	17.7%		
Gender	Female	83	81	164	4.40	0.036
		55.0%	67.5%	60.5%		
	Male	68	39	107		
		45.0%	32.5%	39.5%		
Educational level	Primary school	5	4	9	4.12	0.127
		3.3%	3.3%	3.3%		
	High school	24	31	55		
		15.9%	25.8%	20.3%		
	College/university	122	85	207		
		80.8%	70.8%	76.4%		
Occupation	Healthcare professional	39	8	47	20.26	0.005
		25.8%	6.7%	17.3%		
	Office worker	29	19	48		
		19.2%	15.8%	17.7%		
	Manual laborer	5	5	10		
		3.3%	4.2%	3.7%		
	Unemployed	27	27	54		
		17.9%	22.5%	19.9%		
	Housewife	12	16	28		
		7.9%	13.3%	10.3%		
	Student	16	18	34		
		10.6%	15.0%	12.5%		
	Retired	5	6	11		
		3.3%	5.0%	4.1%		
	Other	18	21	39		
		11.9%	17.5%	14.4%		
Monthly income	Less than 5000 SAR	49	57	106	10.87	0.028
		32.5%	47.5%	39.1%		
	5000 to 10000 SAR	26	22	48		
		17.2%	18.3%	17.7%		
	10001 to 15000 SAR	25	18	43		
		16.6%	15.0%	15.9%		
	15001 to 20000 SAR	33	11	44		
		21.9%	9.2%	16.2%		
	More than 20000 SAR	18	12	30		
		11.9%	10.0%	11.1%		

Table 9 shows that attitude toward varicella zoster vaccination has a statistically significant relation to age (p=0.048) and monthly income (p=0.029). It also shows no statistically significant difference regarding gender, educational level, and occupation. Participants aged 25 to 34 and those earning between 15001 and 20000 SAR were found to have a higher attitude level than others.

**Table 9:** Attitude toward varicella zoster vaccination in Riyadh in association with sociodemographic characteristics Data are represented as N, %, mean ± SD, etc., and include the value at which the p-value is considered significant (p<0.05).

Parameters		Attitude level		Total (N=271)	χ²	p-value*
		High attitude level	Moderate or low attitude level			
Age	18-24	23	33	56	9.59	0.048
		17.8%	23.2%	20.7%		
	25-34	38	24	62		
		29.5%	16.9%	22.9%		
	35-44	23	19	42		
		17.8%	13.4%	15.5%		
	45-54	28	35	63		
		21.7%	24.6%	23.2%		
	55 or more	17	31	48		
		13.2%	21.8%	17.7%		
Gender	Female	73	91	164	1.59	0.207
		56.6%	64.1%	60.5%		
	Male	56	51	107		
		43.4%	35.9%	39.5%		
Educational level	Primary school	5	4	9	0.24	0.889
		3.9%	2.8%	3.3%		
	High school	26	29	55		
		20.2%	20.4%	20.3%		
	College/university	98	109	207		
		76.0%	76.8%	76.4%		
Occupation	Healthcare professional	31	16	47	10.75	0.150
		24.0%	11.3%	17.3%		
	Office worker	18	30	48		
		14.0%	21.1%	17.7%		
	Manual laborer	6	4	10		
		4.7%	2.8%	3.7%		
	Unemployed	25	29	54		
		19.4%	20.4%	19.9%		
	Housewife	13	15	28		
		10.1%	10.6%	10.3%		
	Student	13	21	34		
		10.1%	14.8%	12.5%		
	Retired	4	7	11		
		3.1%	4.9%	4.1%		
	Other	19	20	39		
		14.7%	14.1%	14.4%		
Monthly income	Less than 5000 SAR	45	61	106	10.79	0.029
		34.9%	43.0%	39.1%		
	5000 to 10000 SAR	26	22	48		
		20.2%	15.5%	17.7%		
	10001 to 15000 SAR	14	29	43		
		10.9%	20.4%	15.9%		
	15001 to 20000 SAR	28	16	44		
		21.7%	11.3%	16.2%		
	More than 20000 SAR	16	14	30		
		12.4%	9.9%	11.1%		

Table 10 shows that practice toward varicella zoster vaccination has a statistically significant relation to age (p=0.004) and gender (p=0.012). It also shows a statistically insignificant relation to educational level, occupation, and monthly income. Participants aged 50 or more and of male gender were found to have a better practice level than others.

**Table 10:** Practice toward varicella zoster vaccination in Riyadh in association with sociodemographic characteristics Data are represented as N, %, mean ± SD, etc., and include the value at which the p-value is considered significant (p<0.05).

Parameters		Practice level		Total (N=271)	χ²	p-value*
		Good practice level	Poor practice level			
Age	18-24	15	41	56	15.44	0.004
		29.4%	18.6%	20.7%		
	25-34	9	53	62		
		17.6%	24.1%	22.9%		
	35-44	1	41	42		
		2.0%	18.6%	15.5%		
	45-54	11	52	63		
		21.6%	23.6%	23.2%		
	55 or more	15	33	48		
		29.4%	15.0%	17.7%		
Gender	Female	23	141	164	6.25	0.012
		45.1%	64.1%	60.5%		
	Male	28	79	107		
		54.9%	35.9%	39.5%		
Educational level	Primary school	3	6	9	1.41	0.494
		5.9%	2.7%	3.3%		
	High school	11	44	55		
		21.6%	20.0%	20.3%		
	College/university	37	170	207		
		72.5%	77.3%	76.4%		
Occupation	Healthcare professional	8	39	47	12.19	0.095
		15.7%	17.7%	17.3%		
	Office worker	8	40	48		
		15.7%	18.2%	17.7%		
	Manual laborer	1	9	10		
		2.0%	4.1%	3.7%		
	Unemployed	12	42	54		
		23.5%	19.1%	19.9%		
	Housewife	2	26	28		
		3.9%	11.8%	10.3%		
	Student	10	24	34		
		19.6%	10.9%	12.5%		
	Retired	5	6	11		
		9.8%	2.7%	4.1%		
	Other	5	34	39		
		9.8%	15.5%	14.4%		
Monthly income	Less than 5000 SAR	17	89	106	5.05	0.282
		33.3%	40.5%	39.1%		
	5000 to 10000 SAR	11	37	48		
		21.6%	16.8%	17.7%		
	10001 to 15000 SAR	5	38	43		
		9.8%	17.3%	15.9%		
	15001 to 20000 SAR	9	35	44		
		17.6%	15.9%	16.2%		
	More than 20000 SAR	9	21	30		
		17.6%	9.5%	11.1%		

## Discussion

This study evaluated understandings, behaviors, and perceptions about varicella zoster vaccination within the resident community of the Riyadh First Health Care Cluster in Saudi Arabia. The research data show that participants hold positive vaccination opinions but display an inadequate understanding and application of vaccination practices. This section contrasts the current research findings with previous studies to highlight both complementary and discrepant data on vaccine-related awareness and utilization and then discusses research limitations.

The research revealed that only 15.5% of survey respondents achieved a high level of knowledge of varicella zoster vaccinations, posing serious implications for public vaccination awareness levels. According to Alnasser et al., healthcare providers in Saudi Arabia exhibit gaps in understanding how vaccines work and their safety, which creates conditions for public vaccine skepticism [20]. Data from 40.2% of participants showed moderate knowledge of vaccinations. At the same time, they also displayed essential misunderstandings that match the findings of Clements et al. regarding the need for purposeful educational initiatives to build public understanding of vaccines [21].

Through research findings, the investigators determined that varicella zoster vaccination was received with high acceptance by 47.6% of participants. Similar to the research results of Tseng et al. [22], populations receiving dependable healthcare information demonstrated favorable vaccination attitudes. Further investigations are necessary to study the factors that drive individuals to hold negative attitudes toward vaccinations, as these attitudes are present in 10.7% of respondents. Research shows that vaccine safety concerns significantly influence why people choose not to get vaccinated due to fears of side effects [23]. Side effect concerns were observed in 48.4% of our participants, according to the findings from Kelly et al. when studying Australian public responses to the introduction of the varicella vaccine [23].

Research participants exhibited concerning vaccination behavior, as they adhered to good vaccination practices in only 18.8% of the observed instances. The research results from Hales et al. show divergent data, as good vaccination practices had low actual implementation despite positive attitudes [24]. Data from our study indicate that healthcare providers play a central role in shaping low vaccination levels because they fail to address patient concerns about vaccine safety and necessity, a finding similar to that of Moodley et al. [25]. A lack of communication between healthcare providers and patients regarding vaccines becomes evident through the 75.6% response rate, indicating that many patients did not receive this information from their healthcare providers.

The demographic characteristics influenced how people understood and practiced varicella zoster vaccination. The research data demonstrated that educational status and economic standing act as predictors for different vaccine-related viewpoints. Wu et al. used research to demonstrate that socioeconomic status influences how people learn about vaccinations and their uptake [26]. The population with college degrees in our study highlights that better education would lead to increased vaccine awareness; however, the significant economic differences demonstrate that healthcare information remains limited [26]. The 19.9% unemployment rate among participants exacerbates this inequality, as it diminishes their ability to make informed health choices, according to Levin et al.'s research on socioeconomic factors that influence vaccination practices [27].

The current study has limitations that need acknowledgment. The present study employed a cross-sectional approach to collect knowledge and attitude data. However, it was unable to establish cause-and-effect relationships due to its lack of longitudinal tracking over time. Study participants may introduce reporting bias because they could exaggerate their self-reported vaccination knowledge and practices. The results from a single healthcare cluster in Riyadh cannot be easily applied to other areas in Saudi Arabia or to different cultural settings.

## Conclusions

The data obtained underscores an urgent need for public health interventions in Riyadh to enhance vaccine awareness, correct misconceptions, and increase acceptance of the varicella zoster vaccine. While there is a generally positive attitude toward the vaccine, significant gaps remain between knowledge and practice, highlighting the necessity for immediate action to improve health outcomes related to varicella zoster in the community.

To address the identified gaps, targeted educational initiatives should be implemented that engage healthcare providers and community members. These initiatives must focus on correcting misconceptions and enhancing understanding of the vaccine's benefits. Comprehensive educational interventions throughout the Riyadh community are crucial to strengthening future uptake of the varicella zoster vaccine, promoting overall vaccination, and overcoming persistent barriers to immunization.

## Appendices

**Table 11:** Data collection tool

Arabic (العربية)	English
البيانات الديموغرافية:	Demographic information
1. العمر:	1. What is your age group?
42	42
59	59
79	79
99	99
55 أو أكثر	55 and above
2. الجنس:	2. What is your gender?
أنثى	Female
ذكر	Male
3. المستوى التعليمي:	3. What is your highest level of education?
ابتدائي	Primary school
ثانوي	Secondary school
جامعي/جامعية	College/university
4. المهنة:	4. What is your current occupation?
متخصص في الرعاية الصحية	Healthcare professional
موظف مكتب	Office worker
عامل يدوي	Manual laborer
عاطل عن العمل	Unemployed
ربة منزل	Homemaker
طالب/طالبة	Student
متقاعد	Retired
أخرى (حدد)	Other (please specify): ___________
5. الدخل الشهري (ريال سعودي):	5. What is your monthly income?
أقل من 5000	Less than 5,000 SAR
5000 إلى 10000	5,000-10,000 SAR
10001 إلى 15000	10,001-15,000 SAR
15001 إلى 20000	15,001-20,000 SAR
أكثر من 20000	Above 20,000 SAR
6. ما هو الغرض الأساسي من لقاح الحماق النطاقي؟	6. What is the primary purpose of the varicella zoster vaccination?
الوقاية من جدري الماء والهربس النطاقي	Prevention of chickenpox and shingles
علاج الالتهابات الفيروسية الحالية	Treatment for existing viral infections
تعزيز المناعة العامة	Boosting overall immunity
لا شيء مما سبق	None of the above
7. كم مرة يجب أن يتلقى البالغون جرعة معززة من لقاح الحماق النطاقي للحماية المثلى؟	7. How often should adults receive a booster dose of the varicella zoster vaccine?
كل 5 سنوات	Every 5 years
كل 10 سنوات	Every 10 years
مرة واحدة فقط في العمر	Only once in a lifetime
لا حاجة لجرعات معززة	No booster doses needed
8. هل يمكن إعطاء لقاح الحماق النطاقي أثناء الحمل؟	8. Can varicella zoster vaccination be administered during pregnancy?
نعم، آمن في أي مرحلة	Yes, it is safe during any trimester
نعم، ولكن فقط في الثلث الأول	Yes, but only during the first trimester
لا، ممنوع أثناء الحمل	No, it is contraindicated during pregnancy
لست متأكدًا	Not sure
9. من هو الأكثر عرضة لمضاعفات عدوى الحماق النطاقي؟	9. Which population is most at risk for complications from varicella zoster infection?
الأطفال فقط	Children only
كبار السن فقط	Older adults only
الحوامل، الرضع، وأصحاب المناعة الضعيفة	Pregnant women, infants, and immunocompromised individuals
البالغون الأصحاء فقط	Healthy adults only
10. بالإضافة إلى جدري الماء والهربس النطاقي، يمكن أن يسبب فيروس الحماق النطاقي:	10. In addition to chickenpox and shingles, varicella zoster virus can also cause:
الإنفلونزا	Influenza
الحصبة	Measles
النكاف	Mumps
لا شيء مما سبق	None of the above
11. ما هو المصدر الأساسي للمعلومات حول لقاح الحماق النطاقي بالنسبة لك؟	11. What is the primary source of information for varicella zoster vaccination for you?
متخصصو الرعاية الصحية	Healthcare professionals
الإعلام (التلفزيون، الجرائد، إلخ)	Media (TV, newspapers, etc.)
الإنترنت	Internet
العائلة والأصدقاء	Family and friends
12. ما مدى فعالية لقاح الحماق النطاقي في الوقاية من الهربس النطاقي؟	12. How effective is the varicella zoster vaccine in preventing shingles?
فعال جدًا	Highly effective
فعال بشكل معتدل	Moderately effective
غير فعال كثيرًا	Not very effective
غير فعال على الإطلاق	Not effective at all
13. ما الفئة العمرية التي يُنصح بها بشكل أساسي لأخذ لقاح الحماق النطاقي؟	13. Which age group is the varicella zoster vaccine primarily recommended for?
الأطفال فقط	Children only
البالغون فقط	Adults only
الأطفال والبالغون	Both children and adults
كبار السن فقط	Elderly only
14. هل يمكن لشخص أصيب بجدري الماء في الماضي أن يستفيد من لقاح الحماق النطاقي؟	14. Can a person who has had chickenpox still benefit from the varicella zoster vaccine?
نعم	Yes
لا	No
لست متأكدًا	Not sure
15. ما هي الآثار الجانبية الشائعة للقاح الحماق النطاقي؟ (اختر كل ما ينطبق)	15. What are the common side effects of the varicella zoster vaccine? (Select all that apply.)
ألم أو تورم في مكان الحقن	Pain or swelling at the injection site
حمى	Fever
طفح جلدي	Rash
لا شيء مما سبق	None of the above
16. ما مدى ثقتك في فعالية لقاح الحماق النطاقي؟	16. How confident are you in the effectiveness of varicella zoster vaccination?
واثق جدًا	Very confident
واثق إلى حد ما	Somewhat confident
محايد	Neutral
غير واثق كثيرًا	Somewhat not confident
غير واثق على الإطلاق	Not confident at all
17. إلى أي درجة تثق في المعلومات المقدمة من متخصصي الرعاية الصحية حول لقاح الحماق النطاقي؟	17. To what extent do you trust information provided by healthcare professionals about the vaccine?
أثق تمامًا	Completely trust
أثق إلى حد كبير	Trust to a large extent
محايد	Neutral
لا أثق كثيرًا	Do not trust much
لا أثق على الإطلاق	Do not trust at all
18. ما مدى أهمية مناقشة لقاح الحماق النطاقي أثناء الفحوصات الروتينية؟	18. How important is it for healthcare professionals to discuss varicella zoster vaccination during routine check-ups?
مهم جدًا	Extremely important
مهم	Important
محايد	Neutral
غير مهم كثيرًا	Not very important
غير مهم على الإطلاق	Not important at all
19. ما مدى قلقك من الآثار الجانبية المحتملة للقاح الحماق النطاقي؟	19. How concerned are you about the potential side effects of the vaccine?
قلق جدًا	Very concerned
قلق إلى حد ما	Somewhat concerned
محايد	Neutral
غير قلق كثيرًا	Not very concerned
غير قلق على الإطلاق	Not concerned at all
20. ما مدى تأثير لقاح الحماق النطاقي على صحة المجتمع؟	20. In your opinion, how significant is the impact of varicella zoster vaccination on community health?
تأثير كبير جدًا	Extremely significant
تأثير كبير	Significant
محايد	Neutral
تأثير ضئيل	Insignificant
لا تأثير على الإطلاق	Extremely insignificant
21. ما مدى احتمالية توصيتك بلقاح الحماق النطاقي للعائلة والأصدقاء؟	21. How likely are you to recommend the vaccine to family and friends?
مرجح جدًا	Very likely
مرجح	Likely
محايد	Neutral
غير مرجح	Unlikely
غير مرجح على الإطلاق	Very unlikely
22. إلى أي درجة تعتقد أن لقاح الحماق النطاقي يساهم في تكاليف الرعاية الصحية؟	22. To what extent do you believe varicella zoster vaccination contributes to overall healthcare costs?
يساهم بشكل كبير	Significantly contributes
يساهم	Contributes
محايد	Neutral
لا يساهم كثيرًا	Does not contribute much
لا يساهم على الإطلاق	Does not contribute at all
23. ما مدى أهمية قيام الجهات الصحية الحكومية بالترويج للقاح الحماق النطاقي؟	23. How important is it for government health agencies to promote this vaccine through awareness campaigns?
مهم جدًا	Extremely important
مهم	Important
محايد	Neutral
غير مهم كثيرًا	Not very important
غير مهم على الإطلاق	Not important at all
24. ما مدى ثقتك في شفافية المعلومات المقدمة من شركات الأدوية حول لقاح الحماق النطاقي؟	24. How confident are you in the transparency of information provided by pharmaceutical companies?
واثق جدًا	Very confident
واثق إلى حد ما	Somewhat confident
محايد	Neutral
غير واثق كثيرًا	Somewhat not confident
غير واثق على الإطلاق	Not confident at all
25. ما مدى إلمام الجمهور العام بلقاح الحماق النطاقي؟	25. In your opinion, how well-informed is the general public about varicella zoster vaccination?
ملم جدًا	Very well-informed
ملم	Well-informed
محايد	Neutral
غير ملم كثيرًا	Not very well-informed
غير ملم على الإطلاق	Not well-informed at all
26. هل تلقيت لقاح الحماق النطاقي من قبل؟	26. Have you ever received the varicella zoster vaccine?
لا	No
نعم	Yes
27. إذا كانت الإجابة نعم، هل أكملت الجرعات الموصى بها؟	27. If yes, did you complete the recommended number of doses?
لا	No
نعم	Yes
28. إذا كانت الإجابة لا، ما هي أسباب عدم التطعيم؟ (اختر كل ما ينطبق)	28. If no, what are your reasons for not getting vaccinated? (Select all that apply.)
نقص الوعي	Lack of awareness
مخاوف بشأن سلامة اللقاح	Concerns about vaccine safety
الاعتقاد بأن اللقاح غير ضروري	Belief that the vaccine is unnecessary
التكلفة	Cost
أخرى (حدد)	Other (please specify): ___________
29. هل سبق لك أن طلبت معلومات عن لقاح الحماق النطاقي من مقدمي الرعاية الصحية؟	29. Have you ever actively sought information about the vaccine from healthcare providers?
لا	No
نعم	Yes
30. كم مرة تناقش لقاح الحماق النطاقي مع مقدم الرعاية الصحية الخاص بك؟	30. How frequently do you discuss varicella zoster vaccination with your healthcare provider?
بانتظام	Regularly
أحيانًا	Occasionally
نادرًا	Rarely
أبدًا	Never
